# The Book World of Medicine and Science

**Published:** 1898-09-24

**Authors:** 


### The Book World of Medicine and Science.

Practical Organic Chemistry: The Detection and Properties of some of the More Important Organic Compounds. By Samuel Rideal, D.Sc.London, Examiner in Chemistry Royal College of Physicians, London. Second edition. (London: H. K. Lewis. 1898. Pp. 172. Price 2s. 6d.)

This book has evidently served its purpose well, as the issue of a second edition testified. Under each heading the principal sources and modes of origin of the common organic compounds are given, together with the methods of their identification. Dr. Rideal has not given tables or schemes of analysis, but only such methods of procedure as the student can construct for himself and by which he can satisfy himself that he has mastered the subject fully. The book deals with the detection of various elements in organic bodies, with the organic acids, carbohydrates, organic bases, and neutral substances. It is written especially for students reading for the B.Sc. or Intermediate M.B. examinations of the London University. In this second edition several organic substances recently included in the schedules of various examinations are given, together with a few other compounds of general interest. Alterations brought about by the new edition of the British Pharmacopœia have been noted where necessary. The book is an excellent one for students, and also for those who desire to extend their acquaintance with the subject beyond the limits required by examinations.

The Five Laws of Health. By A. T. Schofield, M.D. (London: The Religious Tract Society. 1898. Price 1d.)

This is one of a series of pamphlets by Dr. Schofield which, under the title "Health at Home," is now being issued by the Religious Tract Society. Dr. Schofield is well known as a writer who is able in a pleasant way to convey hygienic information to the ignorant, and in this pamphlet the necessity for cleanliness, good food, good air, suitable clothing, and proper exercise is enforced simply and concisely. As the publisher's advertisement says, words of counsel are also given for the life and health of the soul.

B. Bradshaw's Dictionary of Bathing Places, Climatic Health Resorts, Mineral Waters, Sea Baths, and Hydropathic Establishments. 1898. With a map and several smaller maps and plans. (London: Kegan Paul, Trench, Trübner, and Co. Price 2s. 6d.)

We have found this a very useful book. A short description is first given of the different classes of mineral waters and of the processes which are adopted at the different health resorts, and this is followed by a few pages of hints to travellers of the ordinary guide book type. Then comes a table of the main diseases for the treatment of which people fly to spas and health resorts, giving under each head the places where they are most effectually treated—or perhaps it would be more proper to say where at the present time it is most fashionable to send them. Then come details as to mode of travel, time, and cost, and finally we reach what forms the great bulk of the work, namely, the dictionary, in which details are given regarding an enormous number of places which are resorted to for one purpose or another in the treatment of disease. We have tested the contents of this book in many ways, and have generally found the information, so far as it goes, correct and up to date. It must be remembered that so great is the number of places mentioned that in regard to many of them the information must be but scanty. Those places which are more commonly visited are, however, treated with considerable fulness. Medical men are expected to be so omniscient in regard to all matters pertaining to all forms of treatment, however fanciful, that they will probably often find this little dictionary a useful work of reference in answering the unexpected and unforeseeable questionings of their patients, for we must confess that many places are mentioned in its pages of the very existence of which we, and we fancy most people, have never heard.

A Dictionary of Diseases and Their Treatment. By a Member of the London Royal College of Physicans. (London: *Chemist and Druggist* Office. Price 3s. 6d. Pp. 183.)

The only preface to this book is the following: "Important notice.—'The Dictionary of Diseases,' written by a member of the London Royal College of Physicians, treats upon every malady afflicting humanity from childhood to the grave, giving much practical advice, with prescriptions, thus at once rendering it an invaluable and indispensable publication in each and every home." Even were venereal diseases, all diseases of the male and female organs of generation, and other diseases not conspicuous by their absence from this work, we do not think it could justly claim either to "treat upon every malady, &c.," or to be a work "for every family." The book would seem to have been written with the object of conveying to the non-medical public a heterogeneous mass of information on drugs, diseases, and means of treatment. As regards any principle (except an alphabetical one) on which it has been put together, none is apparent, but prominence seems to be given to matters involving personal appearance, such as the hair, warts, naevus, corns, the feet, spine, nose, and teeth. These are all discussed at some length, while diseases such as enteritis, dropsy, cretinism, and insanity receive in comparison but casual mention. The cover of this "invaluable and indispensable" work, as well as its introductory notice, and the advertisements contained within its cover, are in complete harmony with its literary style and general intention.

Elements of Histology. By E. Klein, M.D., F.R.S., Lecturer on General Anatomy and Physiology, St. Bartholomew's Hospital, and J. S. Edkins, M.A., M.B., Joint Lecturer and Demonstrator of Physiology, St. Bartholomew's Hospital Medical School. Revised and enlarged edition. (London: Cassell and Co. 1898. Pp. 500; 296 Illustrations.)

A new edition of this book has been called for owing to recent additions to the general knowledge of structural anatomy. In particular it has been found necessary to enlarge and revise the chapters on the structure of the central nervous system and sense organs. These chapters have been practically rewritten, while other parts of the book have also been corrected and amplified. Dr. Edkins, who has rewritten and re-edited the chapters on the brain and medulla and on the alimentary canal, is associated with Dr. Klein as joint author of the latest edition. A number of new illustrations, or illustrations borrowed from other recent works on the subject, have been added. A series of excellent prints from microphotographs are given, which possibly owe some of their exceptional clearness to the excellence of the paper on which they are printed. The book is now as it stands exceedingly full and absolutely up to date. In the chapters on the spinal cord the results of the researches of Golgi, Ramón y Cajal, and Köllinker on "tract cells," and the ramifications of the axon of the ganglion cells are fully described. The methods of Weigert and Pal of staining medullated nerve fibres of the spinal cord, Marchi's method of distinguishing between healthy and degenerated nerve fibres, and Golgi's silver method of staining nerve fibres are alluded to in connection with the important observations which have followed their introduction. The chapters on the organs of special sense contain a clear and minute *résumé* of the most recent discoveries in these matters. The authors are to be congratulated on the issue of this welcome new edition, while we others are to be congratulated on having so lucid and learned a book within our easy reach.

